# Development of maternal and foetal immune responses in cattle following experimental challenge with *Neospora caninum* at day 210 of gestation

**DOI:** 10.1186/1297-9716-44-91

**Published:** 2013-10-03

**Authors:** Paul M Bartley, Frank Katzer, Mara S Rocchi, Stephen W Maley, Julio Benavides, Mintu Nath, Yvonne Pang, Germán Cantón, Jackie Thomson, Francesca Chianini, Elisabeth A Innes

**Affiliations:** 1 Moredun Research Institute, Pentlands Science Park, Bush Loan, Midlothian, EH26 0PZ, Scotland, United Kingdom; 2 Instituto de Ganadería de Montaña (CSIC-ULE), 24346 Grulleros (León), Spain; 3 Biomathematics & Statistics Scotland, James Clerk Maxwell Building, King’s Buildings, Edinburgh EH9 3JZ, Scotland, United Kingdom; 4 Instituto Nacional de Tecnología Agropecuaria (INTA), EEA, Balcarce CC276, Argentina

## Abstract

This study examined the immunological responses of pregnant cattle and their foetuses following an experimental challenge with live *Neospora caninum* tachyzoites at day 210 of gestation. Animals were bled prior to and weekly throughout the experiment and sacrificed at 14, 28, 42 and 56 days post inoculation (dpi). At post mortem examination, samples of lymph nodes and spleen were collected from both dam and foetus for immunological analysis. Subcutaneous (sc) inoculation over the left prefemoral (LPF) lymph node of pregnant cattle at day 210 of gestation, led to the vertical transmission of parasites by 14 dpi, however no foetal deaths were observed in the infected animals. Foetuses from infected dams mounted *Neospora*-specific humoral and cell-mediated immune (CMI) responses by 14 dpi. These responses involved anti-*Neospora* IgG, antigen-specific lymphocyte proliferation, and the production of the cytokines IFN–γ, interleukin (IL)-4 and IL-10. There was also evidence of innate immunity during the response against *Neospora* from infected dams, with statistically significant (*p* < 0.05) increases in mean expression of toll like receptors (TLR)-2 on 56 dpi in maternal spleen, LPF, right prefemoral (RPF), left uterine (LUL) and right uterine (RUL) lymph nodes and TLR-9 in retropharyngeal (RLN), LPF and RPF lymph nodes from 28 dpi. Statistically significant (*p* < 0.05) increases in mean TLR-9 were detected in spleen samples from foetuses of infected dams, compared to the foetuses from control animals. Our results show that vertical transmission of the parasite occurred in all infected dams, with their foetuses showing effective *Neospora*-specific cell mediated, humoral and innate immune responses.

## Introduction

*Neospora caninum* is recognised worldwide as a major cause of abortion and foetal death in farmed ruminants, mainly affecting cattle [1]; though sporadic cases have been reported leading to foetal deaths in sheep [2] and goats [3]. Infection with *Neospora* can occur either through vertical (transplacental) transmission of the parasite from dam to foetus [4], or through the ingestion of oocysts, shed by infected dogs [5] in contaminated feed, water and pasture (horizontal transmission). Current control strategies for bovine neosporosis mainly involve farm management and bio-security practices (reviewed by Dubey et al.) [1].

It has become clear from natural and experimental data that primary *Neospora* infections in cattle can lead to abortions and reproductive losses, with cattle infected with *Neospora* for the first time during pregnancy being 3–7 times more likely to abort than uninfected animals [6,7]. Animals can develop a level of natural immunity to the parasite, as cows previously naturally exposed to *Neospora* are less likely to abort following a secondary infection than pregnant animals with primary infections of the parasite [8]. In addition, multigravidae animals that have aborted due to neosporosis are less likely to abort due to neosporosis during subsequent pregnancies, compared to primigravidae cattle [9]. There is also experimental data [10] that demonstrates that exposure of cattle to *N*. *caninum* prior to pregnancy can protect against the vertical transmission of the parasite following an experimental challenge during mid gestation. However in naturally infected cows that were experimentally challenged on day 70 of gestation, the animals showed protection against foetopathy, but vertical transmission of the parasite still occurred [11]. These results suggest that a protective immune response may be induced that can protect against abortion, however, it may be more difficult to prevent endogenous vertical transmission of the parasite.

The gestational stage and immunological maturity of the foetus at time of infection with *N*. *caninum* are critical in determining the clinical outcome of infections in pregnant cattle [10,12,13]. Infection with *N*. *caninum* during early gestation leads to high levels of foetal mortality [14] and more severe pathology than infection of cattle with *N*. *caninum* at mid and late gestation [15]. While an experimental challenge with the parasite at mid gestation did not result in foetal mortality, it did however result in moderate pathology in the foetal central nervous system (CNS) and within the placenta [12]. The foetuses of cattle challenged at mid gestation were shown to be capable of mounting parasite-specific CMI and humoral responses [16]. Observed differences in the maternal immune response to *N*. *caninum* may have a profound effect on clinical outcome. A study by Bartley et al., [17], showed that in a group of pregnant cows given a similar challenge with *N*. *caninum*, some of the animals aborted their foetuses whereas other did not. These clinical differences could be related to the cell-mediated immune response of the animals to the parasite infection [17].

*Neospora*-specific cell mediated and innate immune responses are likely to be involved in protection against the parasite, in naturally [18] as well as experimentally infected animals [10,13,19,20]. Data from several studies has demonstrated that lymphocyte proliferation and interferon-γ (IFN-γ) responses are involved during an immune response to *N*. *caninum*[11,21-23], and that these responding immune cells tend to be CD4^+^ T lymphocytes [20,24,25]. Less is known regarding the role of innate immune responses during bovine neosporosis. Work by Boysen et al. and Klevar et al. [26,27] reported that natural killer (NK) cells produced IFN-γ during early *Neospora* infection in calves. Work in mice infected with *N*. *caninum* has demonstrated that IFN-γ production is dependent on myeloid differentiation factor 88 signalling, in a mechanism triggered by interleukin-12 (IL-12) production in dendritic cells [28]. Increased toll like receptor (TLR)-2 expression leads to the maturation of antigen presenting cells such as macrophages and NK-cells and pro-inflammatory cytokine production [29]. These data show that the innate immune response may be important in the initiation of immune responses to *N*. *caninum*. Understanding of innate immunity to *N*. *caninum* will help to improve the design of effective vaccines, which rely on the induction of appropriate immune responses.

In this study the humoral, cell mediated and innate immune responses were examined in pregnant cattle and their foetuses experimentally challenged with live *N*. *caninum* (NC1 isolate) tachyzoites on day 210 of gestation. This is of particular interest as a primary exposure of cattle to *N*. *caninum* at late gestation can lead to high levels of transplacental transmission and the birth of persistently infected calves [30], which in turn are at risk of transmitting the parasite to their own offspring. This work will further the understanding of the role of both the maternal and foetal immune responses in cattle following an infection with *N*. *caninum* at late gestation and help to elucidate the mechanisms involved in disease pathogenesis and parasite transmission in bovine neosporosis.

## Materials and methods

### Animals, inoculum and experimental design

Fifteen pregnant cattle all being seronegative for *N*. *caninum*, *Toxoplasma gondii*, Bovine Viral Diarrhoea Virus (BVDV), infectious bovine rhinotracheitis and *Leptospira* spp., were divided into 2 groups (comprising of control cattle (*n* = 4) and animals inoculated with *N*. *caninum* (*n* = 11) with animals being sacrificed at 14, 28, 42 and 56 days post inoculation (dpi) (Table 1), a full description of the animals is given in Benavides et al. [30]. Parasites for the inoculum were prepared as previously described [31]. Briefly, *N*. *caninum* (NC1 isolate) tachyzoites were cultured within Vero cell monolayers. After 4 days the parasites were harvested by scraping the infected cell monolayers and releasing the tachyzoites into the supernatant. The parasites were harvested, counted and adjusted to the required concentration 2.5 × 10^8^/mL in phosphate buffered saline (PBS). The 2 mL dose of parasites was inoculated sc into the animals over the left prefemoral lymph node (LPF) within 1 h of harvesting the tachyzoites from tissue culture. Uninfected Vero cells (5 × 10^7^ per 2 mL dose in PBS) were used to inoculate each control animal over the LPF (Table 1). This dose of Vero cells was used, as it was the equivalent number of cells that was present in the parasite inocula.

**Table 1:** Experimental design

		**Post mortem dpi/n =**			
Group	Inoculum	14	28	42	56
Infected	5 × 10^8^ NC1 Tachyzoites	3	3	3	2
Control	5 × 10^7^ Vero cells	1	1	1	1

All animal procedures complied with the Animals (Scientific Procedures) Act 1986 and were approved by the Moredun Research Institute ethics committee.

### Samples collected at post mortem

At post mortem examination, samples of LPF, right prefemoral lymph node (RPF), left uterine lymph node (LUL), right uterine lymph node (RUL), retropharyngeal lymph node (RLN) and spleen were collected from the dams. Samples collected from the foetuses were hepatic lymph node (HLN), mesenteric lymph node (MLN) and spleen. Tissue samples for immunological assays, cell proliferation and cytokine production were collected into sterile wash buffer comprising Hanks Buffered saline solution (HBSS) supplemented with 2% heat inactivated (∆H) foetal bovine serum (FBS) (Labtech International, Ringmer, UK) and 100 IU/mL penicillin and 50 μg/mL streptomycin (Northumbria Biologicals, Cramlington, UK).

Blood was drawn from dams weekly and at post mortem by jugular venipuncture and from foetuses via the cordal vein, into non-heparinised vacutainer blood collection tubes, and allowed to clot; serum was then separated by centrifugation at 2000 × *g* for 10 min and stored at -20 °C prior to enzyme linked immunosorbent assay (ELISA) analysis of anti-*Neospora* IgG.

Sub-samples of the maternal and foetal lymph nodes and spleen were collected for molecular analysis of innate immune responses. Samples were snap frozen on dry ice at post mortem examination, then stored at -80 °C prior to RNA extraction, cDNA synthesis and analysis by SYBR green quantitative polymerase chain reaction (qPCR).

### Serology

Analysis of anti-*Neospora* IgG was performed using a commercially available ELISA kit (IDEXX, Chalfont St Peter, UK) following the manufacturer’s instructions. Samples were considered positive with a sample/positive (S/P) value of ≥ 0.50. The S/P value was calculated using the optical density (OD) results and applying them to the formulae listed below:

$$\frac{\mathrm{Sample}\;\mathrm{OD}\;\mathrm{Result}–\mathrm{Negative}\;\mathrm{Control}\;\mathrm{OD}\;\mathrm{ResultPositive}\;}{\mathrm{Control}\;\mathrm{OD}\;\mathrm{Result}–\mathrm{Negative}\;\mathrm{Control}\;\mathrm{OD}\;\mathrm{Result}}$$

### Preparation of cells for immunological assays

Single cell suspensions of maternal and foetal lymph node and spleen samples were prepared using the method previously described by Bartley et al. [16]. Briefly; tissues were trimmed to remove excess fat and then chopped into small pieces. These pieces were resuspended in 10 mL of wash buffer and placed in a stomacher bag (Seward Medical, Northampton, UK) and homogenized for 10 s. The resultant cell suspension was decanted through a double thickness of sterile lens tissue into a sterile universal. The cells were washed twice by repeated centrifugation at 260 × *g* before being resuspended at a final concentration of 2 × 10^6^/mL in cell culture medium (CCM) (Iscoves modified Dulbeco’s medium (IMDM) (Gibco, Paisley, UK) supplemented with 10% ∆H FBS and 100 IU/mL penicillin and 50 μg/mL streptomycin).

### Cell proliferation assays

Single cell suspensions of both lymph node and spleen were treated as previously described by Bartley et al. [16]. In brief, equal volumes of cells (2 × 10^6^/mL) and antigen were added in quadruplicate to 96-well round bottom plates (Nunc, Roskilde, Denmark). Water-soluble *N*. *caninum* tachyzoite antigen (NCA) [10] was used at a final protein concentration of 1 μg/mL, the T-cell mitogen concanavalin A (Con A) was used as a positive control at a final protein concentration of 5 μg/mL. Vero cell lysate antigen at 1 μg/mL final protein concentration was used as a control antigen and CCM alone was used as a negative control to determine the background level of cell proliferation. The cultures were incubated at 37 °C in a humidified 5% CO_2_ atmosphere for 5 days. The cultures were pulsed with 18.5 kBq ^3^H Thymidine/well (Amersham Biosciences, Little Chalfont, UK) for the final 18 h, before being harvested onto glass-fibre filters (Wallac, Turku, Finland); the cell associated radioactivity was determined using a microbeta Trilux liquid scintillation counter (Perkin Elmer, Wellesley, MA, USA).

Duplicate wells were prepared for each sample; after 4 days incubation at 37 °C in a humidified 5% CO_2_ atmosphere; cell free supernatants were harvested and stored at -20 °C prior to analysis by ELISA to quantify cytokine production.

### Cytokine responses

The concentrations of bovine cytokines; IFN-γ, IL-4, IL-10 and IL-12 present in the cell free supernatant were determined using commercially available cytokine capture ELISA; antibody pairs (Serotec, Oxford, UK) and serial dilutions of appropriate recombinant cytokines were used to create standard regression curves (Table 2).

**Table 2:** Antibody pairs and standard ranges for bovine cytokines IFN-γ, IL-4, IL-10 and IL-12 ELISA

	**Primary antibody**	**Secondary antibody**	**Standard range**	**Reference**
	**(Working concentration (μg/mL))**			
IFN-γ	CC330 (5 μg/mL)	CC302b (2 μg/mL)	5000 pg/mL –10 pg/mL	
IL-4	CC314 (5 μg/mL)	CC313b (2 μg/mL)	125.00 U/mL -0.488 U/mL	[32]
IL-10	CC318 (5 μg/mL)	CC320b (2 μg/mL)	11 U/mL -0.021 U/mL	[33]
IL-12	CC301 (1.25 μg/mL)	CC326b (1 μg/mL)	86.42 U/mL -0.013 U/mL	[34]

The ELISA method used for all of the cytokines was based on that previously described by Kwong et al. [33]. In brief, 96-well ELISA plates (Greiner, Stonehouse, UK) were coated with a primary capture antibody (50 μL per well) (Table 2) and incubated at room temperature (RT) overnight. The plates were washed five times using PBS supplemented with 0.05% Tween 20 (PBS-T) between each step, with the exception of the final 3, 3’, 5, 5’-tetramethylbenzidine (TMB) – sulphuric acid (H_2_SO_4_) stage. The plates were blocked at room temperature for 1 h with PBS-T supplemented with 3 % bovine serum albumin (BSA). Samples and standards (50 μL each) were added and incubated at RT for 1 h. Plates were then coated with an appropriate secondary biotinylated antibody (Table 2) (diluted in PBS-T supplemented with 1% BSA) (50 μL per well) and incubated at RT for 1 h. Streptavidin- horseradish peroxidiase (HRP) (Dako Cytomation, Glostrup, Denmark) diluted 1:500 in PBS-T 1% BSA (50 μL/well) was added and incubated at RT for 45 min. Colour was developed by the addition of TMB substrate (Insight Biotech. Ltd., Wembley, UK) (100 μL/well) and incubated for 10–15 min in the dark. Reactions were stopped by adding 1 M H_2_SO_4_ (50 μL per well). The plates were read at 450/650 nm using a MRX II plate reader (Dynex, East Grinstead, UK). Doubling dilutions of known quantities of appropriate recombinant Ovine (rOv) or Bovine (rBo) cytokines (rOv IFN-γ, rBo IL-4, rBo IL-10 and rOv IL-12) (Moredun Research Institute, Edinburgh, UK) were used to generate a standard regression curve against which the test sample data was fitted. The rOv cytokines were used as standards for the ELISA as good cross reaction between rOv cytokines and bovine cells and antibodies has been previously demonstrated [34,35].

### Extraction of RNA from maternal and foetal tissues

Samples collected and snap frozen on dry ice at post mortem were processed for RNA extraction as follows: approximately 1 g of frozen tissue was cut into small pieces and placed in a Precelys tissue homogeniser tube (Cepheid, Stretton Derbyshire, UK) containing 1.5 mL TRI reagent (Applied Biosystems, Carlsbad, CA, USA) and homogenised for 50 s at 6500 rpm using a Precelys 24 tissue homogeniser, (Cepheid, Stretton Derbyshire, UK). 700 μL of the resultant homogenate was split into each of two fresh microfuge tubes containing a further 300 μL TRI reagent. The samples were then processed to RNA as per manufacturer’s instructions. The final RNA pellet was resuspended in 200 μL of RNase free water, the concentration of RNA was determined by spectrophotometry (Nanodrop ND1000); the samples were then stored at -80 °C prior to cDNA synthesis and qPCR.

### cDNA synthesis from maternal and foetal RNA samples

Following the manufactures instructions, a commercially available high capacity cDNA reverse transcription kit (Applied Biosystems, Carlsbad, CA, USA) was used to create 2 μg of cDNA per sample. Each reaction (20 μL) contained 2 μL 10× RT buffer, 0.8 μL dNTP (100 mM), 2 μL 10× random primers, 1 μL reverse transcriptase (50 U/μL), 1 μL RNase inhibitor, 3.2 μL DNase/RNase free water and 10 μL RNA (0.2 μg/μL); the reaction conditions for the cDNA synthesis reaction were 10 min at 25 °C, 120 min at 37 °C, 5 min at 80 °C then held at 4 °C. Following reverse transcription the cDNA was diluted to 400 μL in DNase / RNase free water and stored at 4 °C prior to qPCR analysis.

### SYBR green qPCR for glyceraldehyde-3-phosphate dehydrogenase (GAPDH), toll like receptor-2 (TLR) and TLR-9

To determine whether TLR-2 and TLR-9 expression is being up or down regulated during *N*. *caninum* infections we examined the expression of the TLRs in both maternal and foetal tissues at different stages following challenge, using the SYBR green qPCR primers and basic protocol described by Menzies and Ingham [36]. All samples were analysed in triplicate, each reaction (20 μL) consisted of 10 μL (2× Fast SYBR green master mix) (Applied Biosystems, Carlsbad, CA, USA), 1 μL each forward and reverse primer (10 pmol), 4 μL DNase/RNase free water and 4 μL cDNA. Analysis was performed under standard conditions including a dissociation step (ABI prism 7000 using sequence detection software (SDS) (v1.2.3) (Applied Biosystems, Carlsbad, CA, USA). The levels of TLR expression were normalised against a reference gene GAPDH, which has been previously shown to be constitutively expressed in bovine tissues [36].

### Statistical analysis

To take into account the increased variability at higher mean values, the maternal and foetal proliferation (cpm) data and cytokine assay data (IFN-γ) were transformed by logarithmic transformation (base 10) prior to the analysis. The TLR-2 and TLR-9 data were adjusted against GAPDH. Separate linear models were fitted to the maternal and foetal proliferative responses, cytokine responses and TLR expression. Due to lack of replication of the control group at each time point, data on the control group were pooled across all time points to estimate the variability. The models included treatment group (comprising five levels: infected group at each of four time points and the control group) as an explanatory variable. The model assumes normal distribution for the errors on the analysis scale and this was checked using appropriate plots. The overall statistical significance of the treatment group was evaluated using the *F*-statistic. If the *F*-statistic was statistically significant (*p* ≤ 0.05), two-sided p-values for multiple comparisons between the means of the infected group at each time point and the pooled mean of the control group were obtained; these p-values were then adjusted using a False Discovery Rate (FDR) approach [37] to take into account the problems arising from multiple comparisons. The adjusted *p*-value, denoted in this paper as *p*_*f*_, summarises the strength of evidence for there being a genuine difference in a way analogous to a standard *p*-value but allowing multiple testing across different times. Difference in the median maternal serology IgG values between the infected and control group across each time point was assessed using a two sample non-parametric Mann–Whitney test. To take into account multiple comparisons at all time points, the FDR approach as discussed above was used to adjust the p-values. In general, the statistical significance is considered at 5% level (*p*_*f*_ < 0.05), though for some instances where *p* < 0.05 but not *p*_*f*_, both *p* and *p*_*f*_ values are provided. All statistical analyses were carried out using the R software version 2.15.2 (R Development Core Team, 2012).

## Results

### Proliferative responses from lymph nodes and spleen

Maternal

Infected dams showed increased mean levels of antigen-specific proliferation (counts per minute (cpm) on log_10_ scale) (Additional file 1) from most tissues examined, compared to the control animals at all the time points, though for most samples no statistically significant differences were seen. On 14 dpi statistically significantly (*p* = 0.034) increased mean levels of antigen-specific proliferative responses were observed in the spleen samples from infected animals, compared to control animals. At the same time point, significantly lower (*p* = 0.023) mean levels of proliferation were observed in the RPF of infected animals (mean, 2.56; 95% CI, 1.73 to 3.40) compared to control animals (mean, 3.99; 95% CI, 3.16 to 4.82). However, after allowing for multiple testing, the adjusted p-values for these mean comparisons were not statistically significant for either spleen (*p*_*f*_ = 0.137) or RPF (*p*_*f*_ = 0.09).

### Foetal

**Figure 1:**
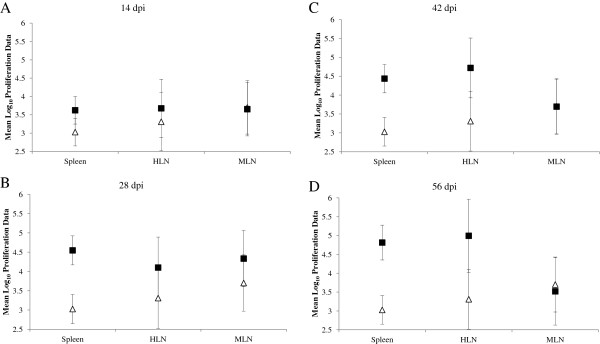
**Foetal lymph node and spleen proliferation responses following stimulation with NCA for 5 days.** Samples of foetal hepatic lymph node (HLN), mesenteric LN (MLN) and and spleen were collected at post mortem examination. Samples were stimulated with NCA for 5 days (37° C in a humidified 5% CO_2_ atmosphere), with 18.5 kBq ^3^H Thymidine / well being added for the final 18 h, before being harvested onto glass-fibre filters. The data were log-transformed (base 10) before analysis by a linear model. The predicted mean responses (counts per minute (cpm) on log_10_ scale) (Infected -■-, Control -∆-) and 95% confidence intervals (error bars) of foetal spleen and lymph node on **(A)** 14 dpi, **(B)** 28 dpi, **(C)** 42 dpi and **(D)** 56 dpi are presented).

The cell proliferation responses from the foetuses are illustrated in Figure 1. Mean levels of antigen-specific proliferation (cpm on log_10_ scale) in the spleen were statistically significantly higher in the infected foetuses on 14 dpi (*p*_*f*_ = 0.032), 28 dpi (*p*_*f*_ < 0.001), 42 dpi (*p*_*f*_ < 0.001) and on 56 dpi (*p*_*f*_ < 0.001) compared to control foetuses. Significantly increased mean levels of proliferation were observed in HLN samples from infected foetuses on 42 dpi (*p*_*f*_ = 0.038) and 56 dpi (*p*_*f*_ = 0.038) compared to the control foetuses. There was no evidence of a difference in the mean levels of antigen-specific proliferation in foetal MLN tissue from either group (Figure 1A-D).

### Cytokine responses of lymph node and spleen

Maternal

#### ***IFN-γ***

Increased mean levels of *Neospora*-specific IFN-γ (ng/mL on log_10_ scale) (Additional file 2) were observed in most maternal lymph nodes and spleen samples from infected dams compared to control animals at all the time points. Statistically significantly higher mean levels of IFN-γ were observed on 14 dpi (*p*_*f*_ = 0.021) in the spleen from infected animals compared to the control animals, while on 56 dpi, mean levels of antigen-specific IFN-γ in spleen samples from infected animals were lower than those seen in the control animals with marginal statistical significance (*p*_*f*_ = 0.060).

#### ***IL-4***

Mean levels of antigen-specific IL-4 were higher in all maternal samples from infected animals compared to the control animals, at all of the time points tested (Additional file 3). Statistically significantly increased mean levels of antigen-specific IL-4 were demonstrated on 14 dpi in the spleen (*p* = 0.028) and LPF (*p* = 0.012) of the infected animals compared to the control animals However, when allowing for multiple testing, the mean differences between treatment groups were not statistically significant for either spleen or LPF (*p* = 0.130, *p* = 0.121 respectively).

#### ***IL-10***

Demonstrable antigen-specific IL-10 was only found in the spleen sample from the control animal on 28 dpi, all other lymph node and spleen samples from the control animals were below the detection threshold of the ELISA. In the infected animals, on 14 dpi demonstrable levels of antigen-specific IL-10 were seen in spleen samples from 2 of 3 animals. On 28 dpi, IL-10 was produced in 3 of 3 samples of spleen and LUL. On 42 dpi (2/3) spleen, (1/3) RUL and RLN samples were shown to be producing IL-10. On 56 dpi, IL-10 was only demonstrable in the LUL from 1 of 2 of the infected animals.

#### ***IL-12***

All the maternal lymph node and spleen samples tested from the control animals were below the detection threshold of the ELISA for antigen-specific IL-12. Demonstrable levels of IL-12 were seen in maternal spleen samples from infected animals on 14 and 28 dpi only.

### Foetal

#### ***IFN-γ***

**Figure 2:**
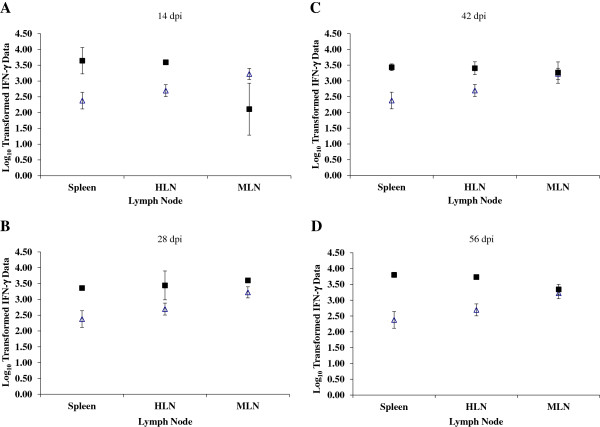
**Foetal lymph node and spleen IFN-γ responses following stimulation with NCA for 4 day.** Foetal hepatic lymph node (HLN), mesenteric LN (MLN) and spleen samples were collected at post mortem examination. Following stimulation with NCA for 4 days (37° C in a humidified 5% CO_2_ atmosphere) cell free supernatants were harvested, ELISA were performed to determine the concentration of IFN-γ (ng/mL) produced. The data were log-transformed (base 10) before analysis by a linear model. The predicted mean responses (ng/mL on log_10_ scale) (Infected -■-, Control -∆-) and 95% confidence intervals (error bars) of foetal spleen and lymph node on **(A)** 14 dpi, **(B)** 28 dpi, **(C)** 42 dpi, **(D)** 56 dpi.

The results from the IFN-γ ELISA are illustrated in Figure 2A-D. Spleen samples from the foetuses in infected dams showed increased mean levels of antigen-specific IFN-γ production (ng/mL on log_10_ scale) compared to the foetuses from control animals on 14, 28, 42 and 56 dpi. These mean differences in IFN-γ levels of the spleen between control and infected foetuses were statistically significant at all time points (*p*_*f*_ = 0.018, *p*_*f*_ = 0.028, *p*_*f*_ = 0.028 and *p*_*f*_ = 0.018 for 14, 28, 42 and 56 dpi respectively) (Figure 2A-D). Samples of HLN and MLN from infected foetuses were generally demonstrated increased mean levels of antigen-specific IFN-γ production compared to the foetuses from the control animals at all time points, though these differences were not statistically significant (Figure 2A-D).

#### ***IL-4***

The infected foetuses showed an increased mean level of production of antigen-specific IL-4 in the spleen compared to the control foetuses on 28, 42 and 56 dpi (*p*_*f*_ = 0.012, *p*_*f*_ = 0.052 and *p*_*f*_ = 0.012 respectively) (Additional file 4). On 14 dpi the mean MLN response from the infected foetuses was significantly higher (*p* = 0.024) compared to the control foetuses. However, this mean difference was not statistically significant once allowance was made for multiple testing (*pf* = 0.114). No statistically significant differences were observed between the control and infected foetuses for mean levels of antigen-specific IL-4 produced by foetal HLN samples.

#### ***IL-10***

All the samples tested for the control foetuses were below the detection threshold of the ELISA. However, demonstrable antigen-specific IL-10 was seen in spleen samples from the infected foetuses at all time points, on 14 dpi one of three foetuses produced IL-10 and on 28 dpi two of three foetuses produced IL-10. On 42 dpi, all infected foetal spleen samples (3 / 3) produced demonstrable antigen-specific IL-10. On 56 dpi, spleen and HLN samples from both infected foetuses produced demonstrable levels of antigen-specific IL-10. The mean levels of IL-10 produced by the foetal samples were comparable with maternal samples (data not shown).

#### ***IL-12***

All the samples tested for control foetuses were below the ELISA detection threshold IL-12 at all time points tested. Spleen samples from infected foetuses (1/3) produced demonstrable levels of IL-12 on 14 dpi. On 28 and 42 dpi, the levels of antigen-specific IL-12 were below detectable levels. However, on 56 dpi, IL-12 was demonstrated from the spleen samples of both infected foetuses. The mean levels of IL-12 produced by the foetal samples were comparable with maternal samples (data not shown).

### Expression of TLR-2 and TLR-9 in lymph node and spleen

Maternal

#### ***TLR-2***

**Figure 3:**
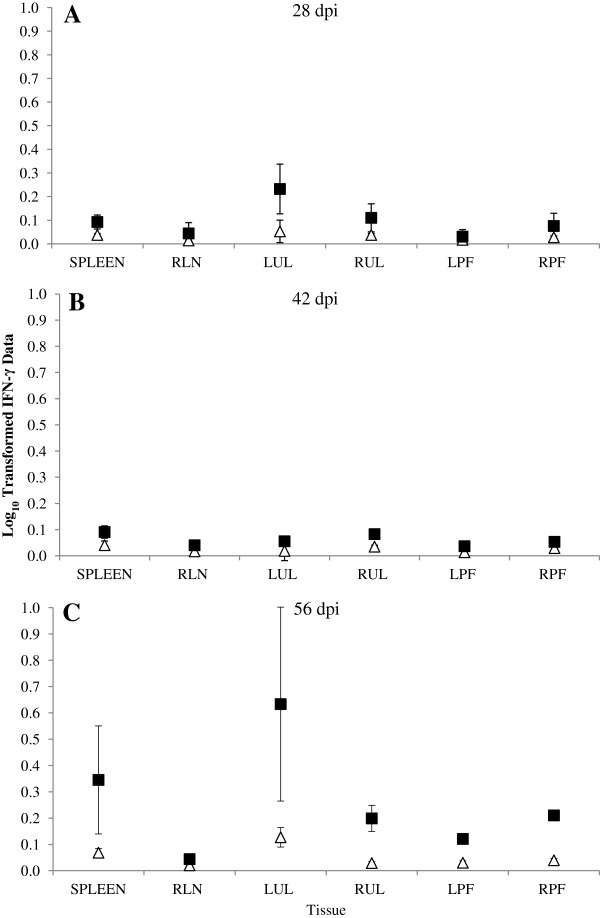
**Maternal lymph node and spleen TLR-2 expression.** Samples of maternal left prefemoral lymph node (LPF), right prefemoral lymph node (RPF), left uterine lymph node (LUL), right uterine lymph node (RUL), retropharyngeal lymph node (RLN) and spleen were collected at post mortem examination and snap frozen on dry ice. RNA was extracted and used to synthesise cDNA. Levels of expression of TLR-2 were examined with data being normalised against GAPDH expression(pg). The predicted mean responses (pg) (Infected -■-, Control -∆-) and 95% confidence intervals (error bars) of maternal spleen and lymph node on **(A)** 28 dpi, **(B)** 42 dpi, **(C)** 56 dpi.

Levels of TLR-2 expression were examined at all time points in both control and infected animals. The infected dams demonstrated increased mean levels of TLR-2 expression (pg) in all tissues compared to the control dams at almost all time points. On 56 dpi, the infected dams demonstrated statistically significantly increased mean levels of TLR-2 expression in the LPF and RPF compared to the control dams (*p*_*f*_ = 0.004 and *p*_*f*_ < 0.001 respectively) (Figure 3C), while the spleen, LUL and RUL samples from the infected animals were all shown to be producing statistically significantly higher mean levels of TLR-2 (*p* = 0.035, *p* = 0.024 and *p* = 0.018 respectively) than the control animals (Figure 3A-C).

#### ***TLR-9***

**Figure 4:**
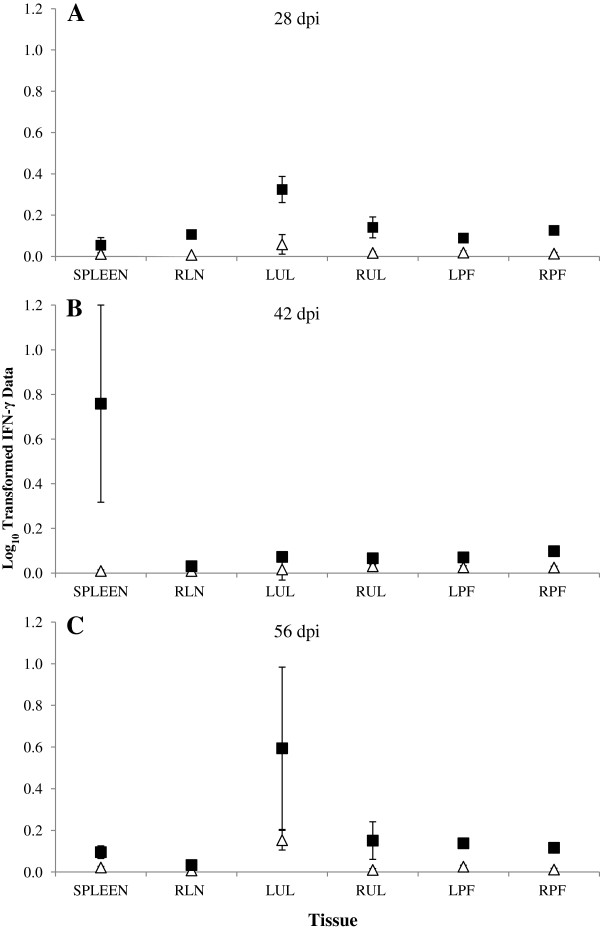
**Maternal lymph node and spleen TLR-9 expression.** Samples of maternal left prefemoral lymph node (LPF), right prefemoral lymph node (RPF), left uterine lymph node (LUL), right uterine lymph node (RUL), retropharyngeal lymph node (RLN) and spleen were collected at post mortem examination and snap frozen on dry ice. RNA was extracted and used to synthesise cDNA. Levels of expression of TLR-9 were examined with data being normalised against GAPDH expression (pg). The predicted mean responses (pg) (Infected -■-, Control -∆-) and 95% confidence intervals (error bars) of maternal spleen and lymph node on **(A)** 28 dpi, **(B)** 42 dpi, **(C)** 56 dpi.

Levels of TLR-9 expression were examined at all time points in both control and infected animals. Infected animals demonstrated increased mean levels of TLR-9 expression (pg) from day 28 onwards in all tissues tested compared to control animals. On 28 dpi statistically significant mean differences were seen between control and infected animals, in RLN (*p* = 0.016), RPF (*p* = 0.042) and LPF (*p* = 0.051) and in spleen (*p* = 0.034) on 42 dpi. While on 56 dpi, LPF (*p*_*f*_ = 0.027) and RPF (*p*_*f*_ = 0.028) from infected animals demonstrated statistically significantly higher mean levels of expression of TLR-9 compared with the control animals (Figure 4A-C).

### Foetal

#### ***TLR-2***

The foetuses from infected animals generally demonstrated increased mean levels of TLR-2 expression (pg) (Additional file 5) in samples of spleen, HLN and MLN compared to the control foetuses. However, these mean differences were not statistically significant. The mean levels of TLR-2 expression seen in the foetal samples were comparable to those observed in the adult samples.

#### ***TLR-9***

When the mean levels of expression of TLR-9 (pg) were examined, statistically significantly increased expression was only observed in the spleen (*p* = 0.025) of the infected foetuses on 42 dpi compared to the control foetuses. However, the mean difference was not statistically significant when adjusted for multiple testing (*p*_*f*_ = 0.101). Generally, the mean expression of TLR-9 of infected foetuses was higher compared to control animals in the spleen, HLN and MLN tissues at all time points. The mean expression of TLR-9 was consistently higher for all samples on 14 dpi, with the spleen demonstrating the highest levels of expression from any of the samples tested (data not shown).

### Serology

### Maternal

**Figure 5:**
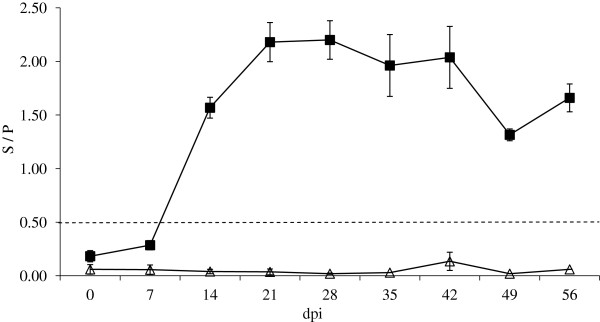
**Maternal serology (IgG ELISA) results.** Blood was drawn from the dams by jugular venipuncture into non-heparinised vacutainer blood collection tubes and allowed to clot. Serum was separated by centrifugation at 2000 × *g* for 10 min and stored at -20 °C prior to analysis of anti-*Neospora* IgG using a commercially available ELISA kit (IDEXX). Samples were considered positive with a sample / positive (S / P) value of ≥ 0.50. The S / P value was calculated using the optical density (OD) results. The estimates of means (Infected -■-, Control -∆-) and standard of means (error bars) are presented.

Following inoculation with *N*. *caninum* tachyzoites all infected dams seroconverted between 7 and 14 dpi (Figure 5). The mean levels of IgG peaked on 21 dpi, the levels of anti-*Neospora* IgG remained elevated throughout the rest of the experimental period. The mean levels of anti-*Neospora* IgG in all control animals remained below the 0.50 cut off throughout. The infected animals demonstrated statistically significantly higher median levels of anti-*Neospora* IgG on 7, 14, 21 and 28 dpi; (*p*_*f*_ = 0.054, *p*_*f*_ = 0.032, *p*_*f*_ = 0.032 and *p*_*f*_ = 0.032 respectively) than the control animals. The median levels of anti-*Neospora* IgG expression in the infected animals remained elevated above the control animals for the remainder of the experiment, though these were not found to be statistically significant.

### Foetal

**Figure 6:**
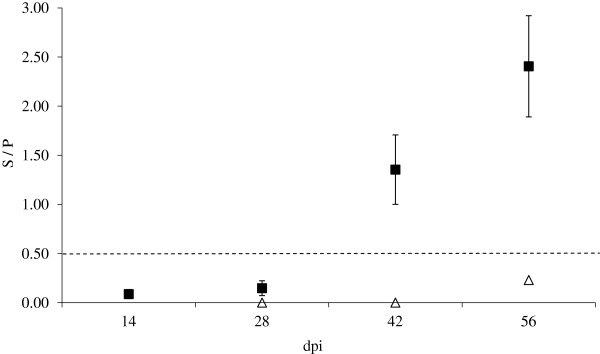
**Foetal serology (IgG ELISA results).** Blood was drawn from the foetuses at the post mortem examination into non-heparinised vacutainer blood collection tubes and allowed to clot. Serum was separated by centrifugation at 2000 × *g* for 10 min and stored at -20 °C prior to analysis of anti-*Neospora* IgG using a commercially available ELISA kit (IDEXX). Samples were considered positive with a sample / positive (S/P) value of ≥ 0.50. The S/P value was calculated using the optical density (OD) results. The estimates of means (Infected -■-, Control -∆-) and standard of means (error bars) are presented.

No anti-*Neospora* IgG was demonstrable in the samples collected from infected foetuses on 14 and 28 dpi. However from 42 dpi onwards all foetuses from infected dams tested positive for anti-*Neospora* IgG (Figure 6). The levels of anti-*Neospora* IgG produced by the infected foetuses were comparable to those seen in the adult infected animals. None of the samples tested from control foetuses were considered positive for anti-*Neospora* IgG as they remained below the 0.50 cut off throughout. No statistical comparisons were made due to the properties of the data.

### Samples collected from control animal

Following post mortem examination, samples from the control animal sacrificed on 14 dpi were shown to be positive by *N*. *caninum* specific ITS1 PCR. Further examination of these samples using primers designed against regions of micro satellite DNA demonstrated that the animal was infected prior to the experiment and the infection was due to a strain of *N*. *caninum* distinct to NC1. As a result of these findings this animal and its foetus were removed from all analysis. Full details of the animal are given in Benavides et al. [30]. This animal was serologically negative for anti-*Neospora* IgG (using IDEXX ELISA) and had no demonstrable antigen specific responses in the cells from the spleen or any of the lymph nodes tested. All the samples tested from the foetus derived from this dam were also immunologically and PCR negative.

## Discussion

At late gestation (day 210), the foetuses are capable of mounting *Neospora* specific cell mediated and humoral immune responses, involving lymphocyte proliferation, antigen-specific IFN-γ, IL-4 and IL-10 production as well as anti-*Neospora* IgG antibodies. This study has also examined the innate immune response during an experimental infection of cattle, showing significantly increased levels of expression of TLR-2 and TLR-9 in a number of maternal and foetal lymphoid tissues including the spleen, uterine and pre-femoral lymph nodes during the course of the infection.

### Maternal CMI responses

Following challenge there were significant increases in cellular proliferation as well as IFN-γ and IL-4 production on 14 dpi in the spleen and peripheral lymph nodes of infected dams and their foetuses, The presence of a CMI response by 14 dpi correlates well with the observation of a circulating parasitaemia observed in all infected dams between 8 – 14 dpi [30], thus providing a source of antigen for immunological priming to take place. The presence of parasites and parasite antigens is likely to lead to the rapid initiation of an immune response. Our findings agree with work by Rosbottom et al. [38] where pregnant cattle experimentally challenged with *N*. *caninum* on day 210 of gestation demonstrated increased numbers of CD4+ T-cells and increased expression of IFN-γ and IL-4 mRNA in PBMC, 1–2 weeks following challenge.

### Toll like receptors

Our study shows that TLR-2 and TLR-9 are up regulated in the spleen and lymph nodes of infected animals and their foetuses during *N*. *caninum* infection. Significant increases in the levels of TLR-2 were not seen in maternal tissues until 56 dpi and TLR-9 on 28 dpi. This is later than we would have expected, as much of the current data regarding TLR function shows they are involved in the initiation and activation of immune responses. Work by Werling et al. [39] in cattle has shown strong TLR-2 signalling to be associated with monocytes and other antigen presenting cells (APC), while TLR-9 signalling was associated with bovine dendritic cells (DC) and B-cells. Similar finding were observed in mice by where TLR-2 was associated with APC maturation and pro-inflammatory cytokine production [28,29]. Cantón et al. [40] observed large numbers of phagocytic cells (in particular macrophages) in resolving lesions in the placenta of infected animals from this experiment from 42 dpi onwards, While on 42 dpi Benavides et al. [30] described lesions in foetal lung and liver characterised by the infiltration of macrophages, lymphocytes and plasma cells. The presence of these cells in the circulatory system may account for the increased levels of TLR-2 expression seen in the spleens of the infected compared to control animals. The increase in TLR-9 expression may be a consequence of increased B-cell activity following the sero-conversion of the infected animals and the production of anti-*Neospora* IgG.

### Humoral immune responses

Infected dams and their foetuses developed strong anti-*N*. *caninum* humoral responses following experimental challenge with the parasite, which continues to suggest a role for antibodies in a protective immune response [41]. Antibody responses were seen in the infected dams from 7 dpi onwards, this coincided with high levels of circulating parasites, however in the foetuses anti-*Neospora* antibodies were not observed until 42 dpi. The delay in the generation of a foetal humoral response could be a consequence of the low numbers of parasites crossing the placenta and actively invading the foetuses, until 28 dpi all foetal tissues were PCR negative [30] Though antibodies against *N*. *caninum* have not been shown to have a definitive role in protection; it is widely believed that they are involved in extracellular tachyzoite neutralisation. Work by Eperon et al. [42] demonstrated increased susceptibility to infection with *N*. *caninum* of B-cell deficient C57BL/6 μMT mice. While work on the closely related parasite *Toxoplasma* has demonstrated roles for antibodies in parasite killing by large granular lymphocytes [43] and in opsonisation and intracellular killing of parasites by mononuclear phagocytes [44].

### Foetal immune response

Due to the nature of the ruminant cotyledonary placentae (syndesmochorial placentation), any immune responses detected in the foetus are likely to be induced by an active infection in utero [45]. Under normal circumstances a cotyledonary placentae does not allow the transfer of maternal immune factors including antibodies and cytokines. During our study foetal cell mediated immune responses were detected at 14 dpi, which suggests that even during the early stages of the infection the parasites crossing the placenta are being dealt with effectively by the foetal immune response, before parasite induced pathology can occur The mild placental pathology being observed, may also be due to there being fewer parasites multiplying in the foetus, therefore there are fewer parasites reinvading the placenta. A reason for this may be that when the parasites reach the placenta they initiate a local maternal immune response. During our experiment increased levels of proliferation and IFN-γ production were seen in the uterine lymph nodes of infected animals compared to the controls at 14 dpi. This may in the first instance limit parasite establishment and multiplication reducing the severity of necrosis and pathology in the tissues around the placenta [45], allowing for more efficient parasite clearing. Coupled with the developing foetal immunity could potentially lead to the scarcity of lesions containing parasite antigen found in the CNS of the infected foetuses. A previous experimental study in cattle [46] showed comparable results to this current study; where cattle infected with *N*. *caninum* (NC Liverpool) at late gestation showed no foetal mortality, though mild placental pathology was observed and parasite DNA was only found sporadically in foetal brain, lung and skeletal muscle.

There is still limited information regarding the development of foetal immune responses during bovine *Neospora* infections. Following infection at early gestation (Day 70 of gestation) [17] showed that no *Neospora*-specific CMI responses were generated, though the foetuses were capable of lymphocyte proliferation and IFN-γ, IL-4, IL-10 and IL-12 production following mitogenic stimulation with Con A from day 84 of gestation onwards, however by mid gestation the foetal immune system is more capable of mounting antigen specific humoral and cell-mediated immune responses [45]. Experimental infections in cattle with *Neospora* at mid gestation (Day 140 of gestation) have shown significant increases in IFN-γ, IL-10 and tumour necrosis factor (TNF) expression [19] and *Neospora*-specific CMI and humoral responses [16] in infected foetuses. While, Andrianarivo et al. [21] showed *Neospora*-specific immune responses in foetal PBMC at around day 220 of gestation.

During our experiment antigen-specific immune responses were present in the foetuses on 14 dpi demonstrating that parasites crossed the placenta, infected the foetuses and induced an immune response. The numbers of parasites in foetal tissues is likely to be very low, as no positive PCR results were seen at 14 dpi [30]. The foetal spleen processes large volumes of blood, this may result in it potentially trapping parasites as well as circulating leukocytes, allowing more rapid presentation of parasite antigens leading to proliferation and cytokine production before the HLN and MLN have started to respond. The presence of parasite DNA and strong cell mediated immune responses being found in all foetuses would suggest that though the parasite crossed the placenta, the foetal immune response was sufficiently robust to control the parasite resulting in the survival of all infected foetuses to the end of the experimental period.

## Conclusions

The results from this study have demonstrated that following an experimental sc challenge of pregnant cattle with live *N*. *caninum* tachyzoites on day 210 of gestation; both dams and foetuses mounting *Neospora*-specific cell mediated, humoral and innate responses. These results show that the stage of gestation is important to disease outcome, with the increasing immunological maturity of the foetus limiting the clinical severity of the infection compared to *Neospora* infections occurring earlier in gestation. The results show that infections with *Neospora* at late gestation may lead to congenitally infected but otherwise clinically normal calves [30]. Experimental challenges of pregnant cattle with *N*. *caninum* tachyzoites allows the detailed study of the host – parasite relationship in bovine neosporosis in a controlled environment, thus improving our understanding of the pathogenesis of the disease.

## Competing interests

The authors declare that they have no competing interests.

## Authors’ contributions

PMB, FK, JB and EAI made substantial contributions to the conception and design. PMB, MSR, JT, YP, GC, SWM, FC and EAI were involved in the acquisition of data. PMB and MN were involved in the analysis of the data. PMB, FK, MN and EAI have been involved in the drafting and critical review of the manuscript. All authors read and approved the final manuscript.

## Supplementary Material

Additional file 1 **Mean Log**_**10 **_**proliferation data from maternal lymph node and spleen samples following stimulation with NCA for 5 days.** Mean Log_10_ proliferation data from maternal lymph node and spleen samples following stimulation with NCA for 5 days. Samples of maternal lymph node and spleen were collected at post mortem examination. Samples were stimulated with NCA for 5 days (37 °C in a humidified 5% CO_2_ atmosphere), with 18.5 kBq ^3^H Thymidine / well being added for the final 18 h, before being harvested onto glass-fibre filters. The data was Log_10_ transformed before analysis by a linear model. (A) 14 dpi, (B) 28 dpi, (C) 42 dpi, (D) 56 dpi. Infected ■, Control ∆ (Error Bars = upper (U) & lower (L) 95% confidence intervals (CI)). Click here for file

Additional file 2 **Mean Log**_**10 **_**transformed IFN-γ data from maternal lymph node and spleen samples following stimulation with NCA for 4 days.** Mean Log_10_ transformed IFN-γ data from maternal lymph node and spleen samples following stimulation with NCA for 4 days. Maternal lymph node and spleen samples were collected at post mortem examination. Following stimulation with NCA for 4 days (37 °C in a humidified 5% CO_2_ atmosphere) cell free supernatants were harvested, ELISA were performed to determine the concentration of IFN-γ produced. The data was Log_10_ transformed before analysis by a linear model. (A) 14 dpi, (B) 28 dpi, (C) 42 dpi, (D) 56 dpi. Infected ■, Control ∆ (Error Bars = U & L 95% CI). Click here for file

Additional file 3 **Concentration of IL-4 in maternal lymph node and spleen samples following stimulation with NCA for 4 days.** Concentration of IL-4 in maternal lymph node and spleen samples following stimulation with NCA for 4 days. Maternal lymph node and spleen samples were collected at post mortem examination. Following stimulation with NCA for 4 days (37 °C in a humidified 5% CO_2_ atmosphere) cell free supernatants were harvested, ELISA were performed to determine the concentration of IL-4 produced. (A) 14 dpi, (B) 28 dpi, (C) 42 dpi, (D) 56 dpi. Infected ■, Control ∆ (Error Bars = U & L 95% CI). Click here for file

Additional file 4 **Concentration of IL-4 in foetal lymph node and spleen samples following stimulation with NCA for 4 days.** Concentration of IL-4 in foetal lymph node and spleen samples following stimulation with NCA for 4 days. Foetal lymph node and spleen samples were collected at post mortem examination. Following stimulation with NCA for 4 days (37 °C in a humidified 5% CO_2_ atmosphere) cell free supernatants were harvested, ELISA were performed to determine the concentration of IL-4 produced. (A) 14 dpi, (B) 28 dpi, (C) 42 dpi, (D) 56 dpi. Infected ■, Control ∆ (Error Bars = U & L 95% CI). Click here for file

Additional file 5 **Levels of expression of TLR-2 in foetal spleen, HLN and MLN samples.** Levels of expression of TLR-2 in foetal spleen, HLN and MLN samples. Samples of foetal lymph node and spleen were collected at post mortem examination and snap frozen on dry ice. RNA was extracted and used to synthesise cDNA. Levels of expression of TLR-2 were examined with data being normalised against GAPDH expression, results are expressed in pg. **(A)** 28 dpi, **(B)** 42 dpi, **(C)** 56 dpi. Infected ■, Control ∆ (Error Bars = U & L 95% CI). Click here for file
