# Supplementary material for: Development of maternal and foetal immune responses in cattle following experimental challenge with Neospora caninum at day 210 of gestation
Source: Vet Res. 2013 Oct 3;44(1):91. doi: 10.1186/1297-9716-44-91 (PMC3851480; doi:10.1186/1297-9716-44-91)
Supplement: Additional file 1 — Mean Log10 proliferation data from maternal lymph node and spleen samples following stimulation with NCA for 5 days. Mean Log10 proliferation data from maternal lymph node and spleen samples following stimulation with NCA for 5 days. Samples of maternal lymph node and spleen were collected at post mortem examination. Samples were stimulated with NCA for 5 days (37 °C in a humidified 5% CO2 atmosphere), with 18.5 kBq 3H Thymidine / well being added for the final 18 h, before being harvested onto glass-fibre filters. The data was Log10 transformed before analysis by a linear model. (A) 14 dpi, (B) 28 dpi, (C) 42 dpi, (D) 56 dpi. Infected ■, Control ∆ (Error Bars = upper (U) & lower (L) 95% confidence intervals (CI)). [file 1297-9716-44-91-S1.doc]

Additional File 1

Mean Log_10_ proliferation data from maternal lymph node and spleen samples following stimulation with NCA for 5 days.

**14 dpi**


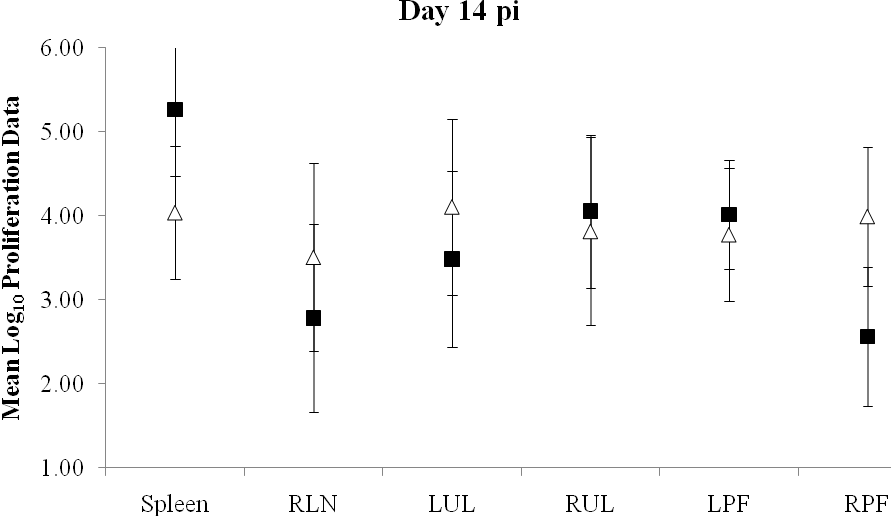


**28 dpi**


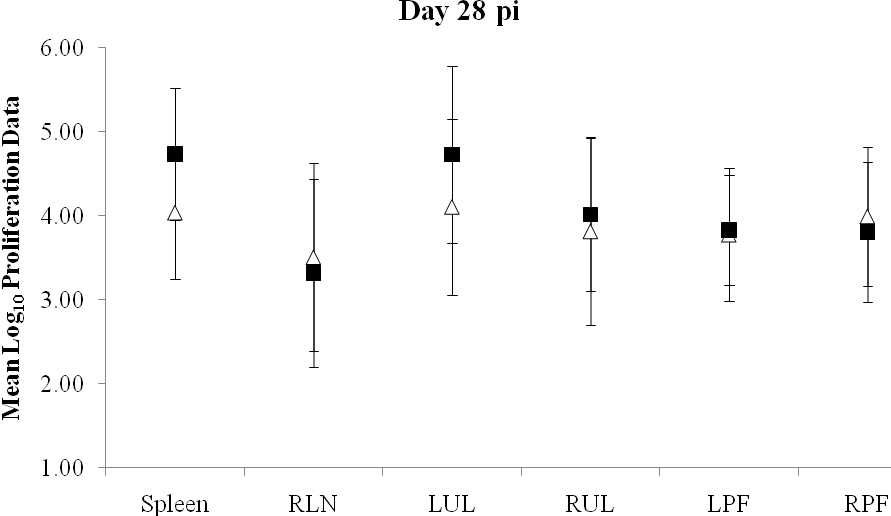


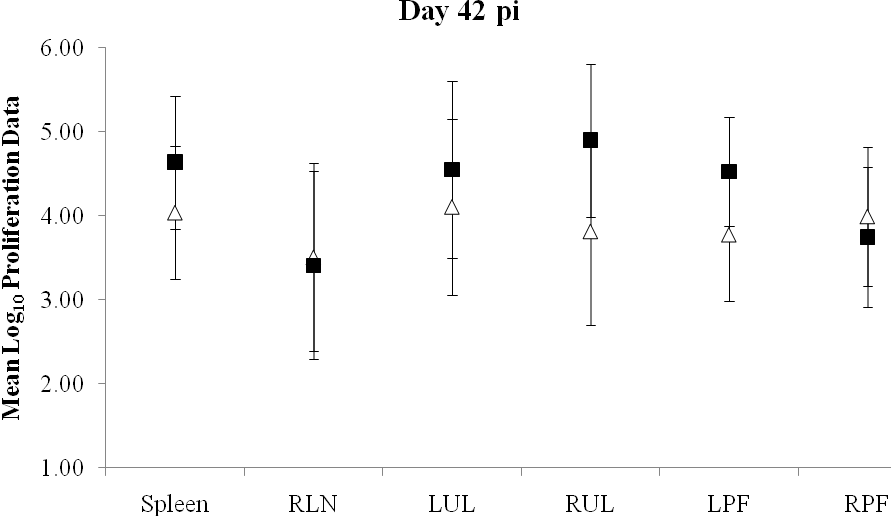


**42 dpi**

**56 dpi**


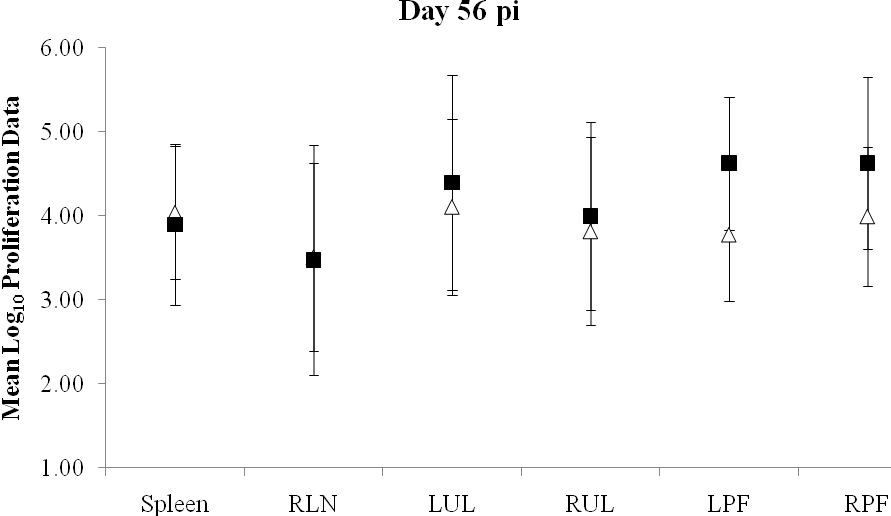


Error bars ± upper and lower 95% confidence intervals

-△- Control -◼- Infected
