# Supplementary material for: Development of maternal and foetal immune responses in cattle following experimental challenge with Neospora caninum at day 210 of gestation
Source: Vet Res. 2013 Oct 3;44(1):91. doi: 10.1186/1297-9716-44-91 (PMC3851480; doi:10.1186/1297-9716-44-91)
Supplement: Additional file 2 — Mean Log10 transformed IFN-γ data from maternal lymph node and spleen samples following stimulation with NCA for 4 days. Mean Log10 transformed IFN-γ data from maternal lymph node and spleen samples following stimulation with NCA for 4 days. Maternal lymph node and spleen samples were collected at post mortem examination. Following stimulation with NCA for 4 days (37 °C in a humidified 5% CO2 atmosphere) cell free supernatants were harvested, ELISA were performed to determine the concentration of IFN-γ produced. The data was Log10 transformed before analysis by a linear model. (A) 14 dpi, (B) 28 dpi, (C) 42 dpi, (D) 56 dpi. Infected ■, Control ∆ (Error Bars = U & L 95% CI). [file 1297-9716-44-91-S2.doc]

Additional File 2

Mean Log_10_ transformed IFN-γ data from maternal lymph node and spleen samples following stimulation with NCA for 4 days.


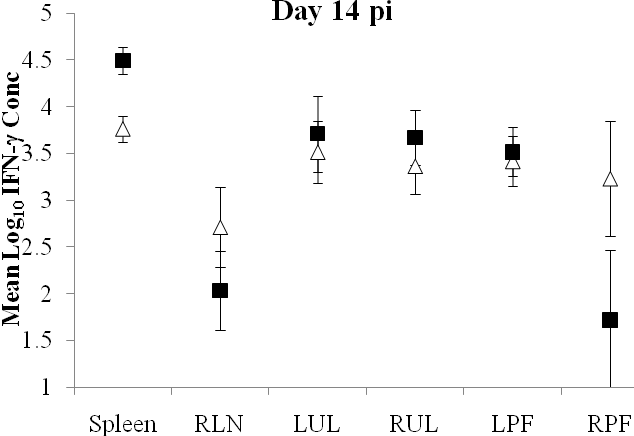


**14 dpi**

**28 dpi**


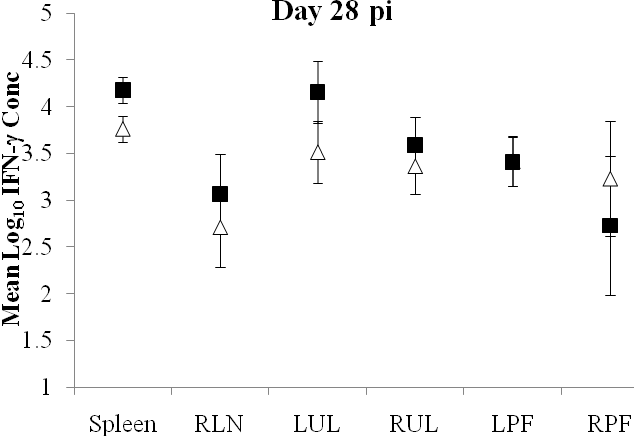


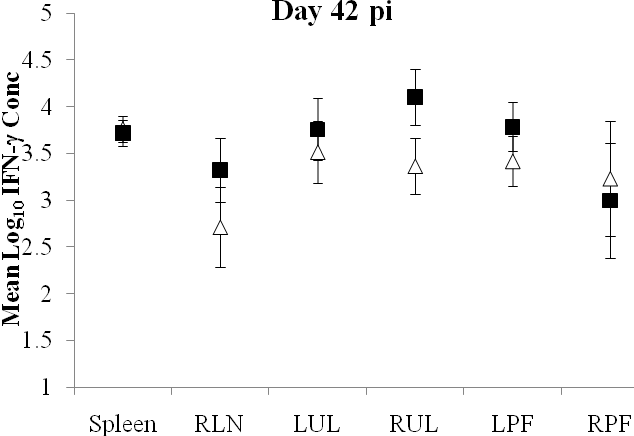


**42 dpi**

**56 dpi**


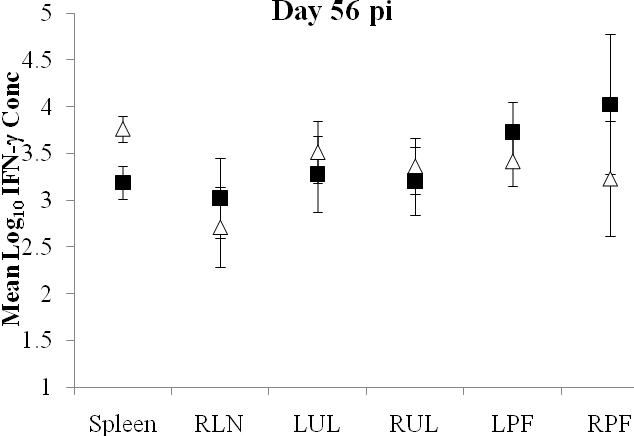


△- Control ◼ - Infected

Error bars ± upper and lower 95% confidence intervals
