# Supplementary material for: Development of maternal and foetal immune responses in cattle following experimental challenge with Neospora caninum at day 210 of gestation
Source: Vet Res. 2013 Oct 3;44(1):91. doi: 10.1186/1297-9716-44-91 (PMC3851480; doi:10.1186/1297-9716-44-91)
Supplement: Additional file 4 — Concentration of IL-4 in foetal lymph node and spleen samples following stimulation with NCA for 4 days. Concentration of IL-4 in foetal lymph node and spleen samples following stimulation with NCA for 4 days. Foetal lymph node and spleen samples were collected at post mortem examination. Following stimulation with NCA for 4 days (37 °C in a humidified 5% CO2 atmosphere) cell free supernatants were harvested, ELISA were performed to determine the concentration of IL-4 produced. (A) 14 dpi, (B) 28 dpi, (C) 42 dpi, (D) 56 dpi. Infected ■, Control ∆ (Error Bars = U & L 95% CI). [file 1297-9716-44-91-S4.doc]

Additional file 4.

Concentration of IL-4 in foetal lymph node and spleen samples following stimulation with NCA for 4 days

**14 dpi**


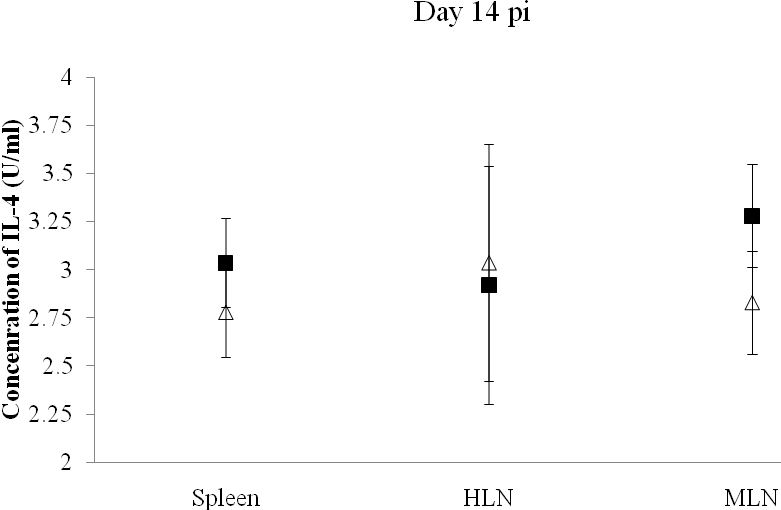


**Conc of IL-4 (U / mL)**

**28 dpi**


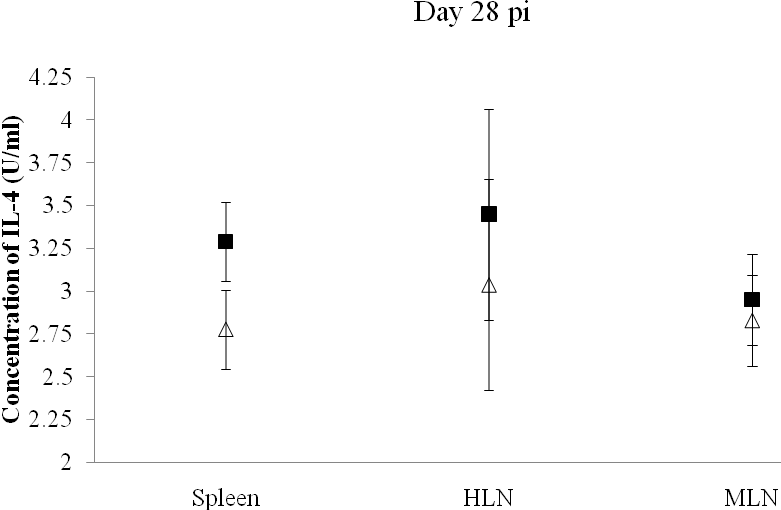


**Conc of IL-4 (U / mL)**


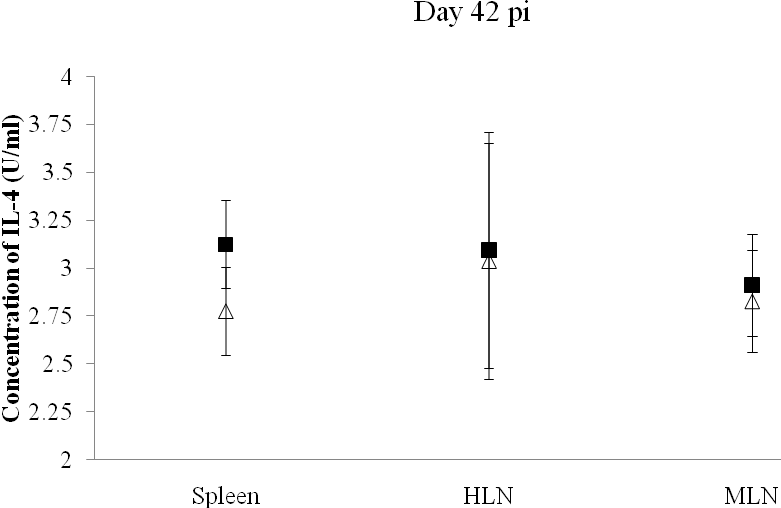


**Conc of IL-4 (U / mL)**

**42 dpi**

**56 dpi**


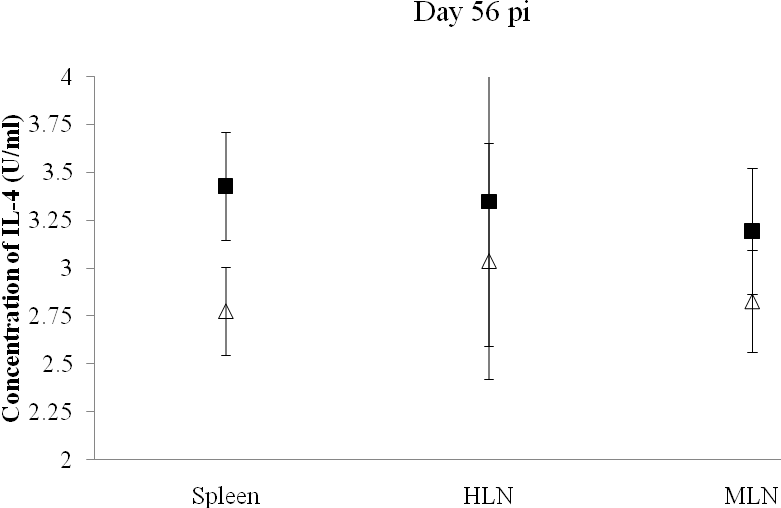


**Conc of IL-4 (U / mL)**

△- Control ◼ - Infected

Error bars ± upper and lower 95% confidence intervals
