# Supplementary material for: Development of maternal and foetal immune responses in cattle following experimental challenge with Neospora caninum at day 210 of gestation
Source: Vet Res. 2013 Oct 3;44(1):91. doi: 10.1186/1297-9716-44-91 (PMC3851480; doi:10.1186/1297-9716-44-91)
Supplement: Additional file 5 — Levels of expression of TLR-2 in foetal spleen, HLN and MLN samples. Levels of expression of TLR-2 in foetal spleen, HLN and MLN samples. Samples of foetal lymph node and spleen were collected at post mortem examination and snap frozen on dry ice. RNA was extracted and used to synthesise cDNA. Levels of expression of TLR-2 were examined with data being normalised against GAPDH expression, results are expressed in pg. (A) 28 dpi, (B) 42 dpi, (C) 56 dpi. Infected ■, Control ∆ (Error Bars = U & L 95% CI). [file 1297-9716-44-91-S5.doc]

Additional file 5.

Levels of expression of TLR-2 in foetal spleen, HLN and MLN samples.

**14 dpi**


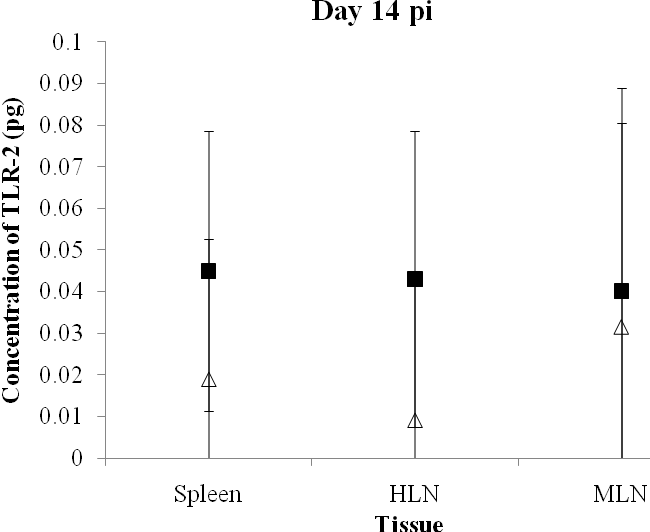


**28 dpi**

**Tissue**

**Tissue**


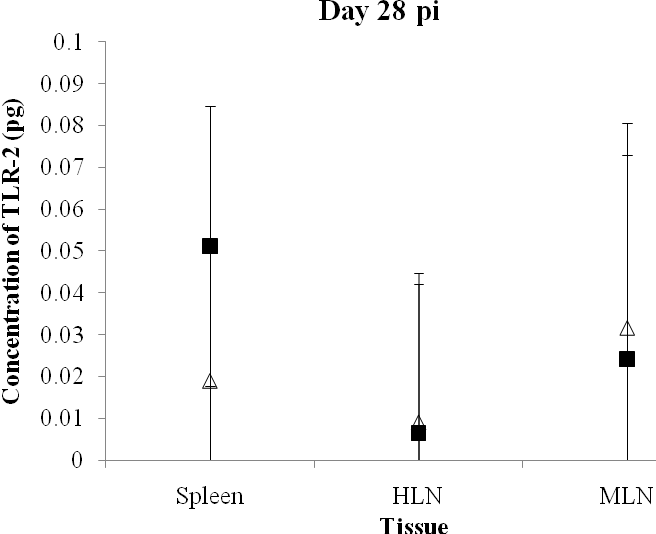


**Tissue**

**Tissue**


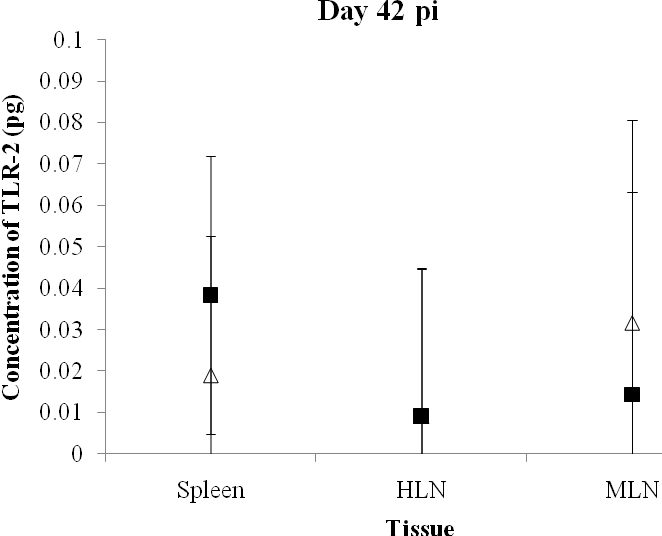


**42 dpi**

**56 dpi**

**Tissue**


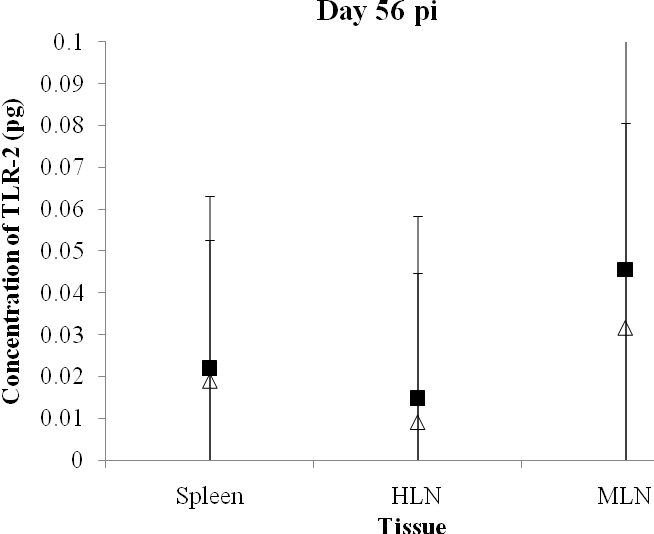


**Tissue**

△- Control ◼ - Infected

Error bars ± upper and lower 95% confidence intervals.
